# Mitochondrial Haplogroups and Polymorphisms Reveal No Association with Sporadic Prostate Cancer in a Southern European Population

**DOI:** 10.1371/journal.pone.0041201

**Published:** 2012-07-17

**Authors:** María Jesús Álvarez-Cubero, María Saiz Guinaldo, Luís Javier Martínez-González, Juan Carlos Álvarez Merino, José Manuel Cózar Olmo, José Antonio Lorente Acosta

**Affiliations:** 1 Laboratory of Genetic Identification, Legal Medicine and Toxicology Department, Facultad de Medicina, Universidad de Granada, Granada, Spain; 2 GENYO (Pfizer-University of Granada-Andalusian Government Centre for Genomics and Oncological Research), Granada, Spain; 3 Service of Urology, University Hospital Virgen de las Nieves, Granada, Spain; Ohio State University Medical Center, United States.of America

## Abstract

**Background:**

It is known that mitochondria play an important role in certain cancers (prostate, renal, breast, or colorectal) and coronary disease. These organelles play an essential role in apoptosis and the production of reactive oxygen species; in addition, mtDNA also reveals the history of populations and ancient human migration. All these events and variations in the mitochondrial genome are thought to cause some cancers, including prostate cancer, and also help us to group individuals into common origin groups. The aim of the present study is to analyze the different haplogroups and variations in the sequence in the mitochondrial genome of a southern European population consisting of subjects affected (n = 239) and non-affected (n = 150) by sporadic prostate cancer.

**Methodology and Principal Findings:**

Using primer extension analysis and DNA sequencing, we identified the nine major European haplogroups and CR polymorphisms. The frequencies of the haplogroups did not differ between patients and control cohorts, whereas the CR polymorphism T16356C was significantly higher in patients with PC compared to the controls (p = 0.029). PSA, staging, and Gleason score were associated with none of the nine major European haplogroups. The CR polymorphisms G16129A (p = 0.007) and T16224C (p = 0.022) were significantly associated with Gleason score, whereas T16311C (p = 0.046) was linked with T-stage.

**Conclusions and Significance:**

Our results do not suggest that mtDNA haplogroups could be involved in sporadic prostate cancer etiology and pathogenesis as previous studies performed in middle Europe population. Although some significant associations have been obtained in studying CR polymorphisms, further studies should be performed to validate these results.

## Introduction

Prostate cancer is one of the most prevalent cancers diagnosed in men. Prostate cancer incidence is characterised by a large geographic variability, ranging from few cases (approximately 4–7 per 100,000) in Asian countries to 70–100 cases per 100,000 in Nordic European countries and North America. In Italy and Spain, the rates are rather low in comparison with those observed in other Western countries and are the lowest among the European Union (EU) countries [1], [2]. However, few conclusive studies have been performed with regard to the genetics of this cancer. Some linkage studies [3] ascribe an important role to genes such as ELAC2 (elaC homolog 2 (E. coli)) at 17q, RNASEL at 1q24–25 (Hereditary Prostate Cancer gene 1 (HPC1)) [4], and MSR1 (macrophage scavenger receptor 1) at 8p22, which contain inactivating mutations in affected members in at least one prostate cancer family. Nevertheless, other studies have not confirmed the associations seen with some of these genes. This is the case with the lack of association between prostate cancer and the RNASEL (2′, 5′-Oligoadenylate-dependent RNase L) gene in a Swedish population [5]. The situation is even more complicated in sporadic prostate cancer where, because of its genetic heterogeneity, it has been suggested that many gene loci, rather than a single specific gene, are involved in the predisposition to this cancer [6]. Two different types of mutations can be found in cancer. Somatic mutations occur in a single cell in developing somatic tissue. The generation of reactive oxygen species (ROS) causes mutations in mitochondrial of somatic cells. In contrast, germinal mutations occur in the germ line and can be passed on to the next generation, as in this study [7]. Mutations in mtDNA (mitochondrial DNA) have been shown to fulfil all of the criteria required for pathogenic mutations causing prostate cancer [8]. Some of the mutations are in the COI (cytochrome c oxidase) [8] or COX7A2 (cytochrome c oxidase subunit VIIa polypeptide 2) [9] gene, and some mutations are directly related to known haplogroups involved in the association between mtDNA variants and complex diseases such as renal and prostate cancer [10]. It has been suggested that mtDNA mutations can be separated into two types: adaptative and tumorigenic (non-adaptative). Adaptative mtDNA mutations are milder mutations observed in different populations [11]. Tumorigenic mutants include mutations such as heteroplasmic insertions and deletions [11]. Mutations in various types of cancers have been observed in both the non-coding and coding regions of mtDNA, but the majority of the mutations identified have been described in the D-loop region (non-coding region). Deletions, insertions in the D-loop region and transitions have been observed in breast, hepatocellular and colorectal cancer mtDNA deletions such as mtDNA4977 have been identified in prostate cancer [12]–[14] and even some mtDNA mutations are related with increased serum PSA[15], [16].

The specific difficulties in understanding the causes of prostate cancer are due to the heterogeneous nature of the disease, its unknown etiology, and the fact that many of the genes involved have multiple variations among populations, are greatly affected by environmental factors among populations, and a large role for environmental effects [17]–[19].

The aim of the present study was to compare the frequencies of mtDNA haplogroups and CR (control region) polymorphisms in 239 patients with sporadic prostate cancer to those in 150 healthy controls in Southwest Europe, as previously done in Korea [20] and Middle European Caucasians [21].

## Results

The nine major European mtDNA haplogroups and CR polymorphisms were analyzed in whole blood samples of 239 patients with sporadic prostate cancer and were compared to 150 control subjects without any familial clinical history of the pathology. Furthermore, controls were confirmed by normal PSA values with blood levels below 4 ng/ml, as well as normal rectal touch. The clinical characteristics of the patients and controls are shown in Table 1.

**Table 1:** Characteristics of the study population.

	Patients withprostate cancer	Controls
	n = 239	n = 150
**Mean (SD** 1 **) age (years)**	66.9 (7.74)	70.2 (8.39)
**Stage**		
**A**	n = 9 (3.76%)	n.a.
**B**	n = 145 (60.67%)	n.a.
**C**	n = 49 (20.50%)	n.a.
**D**	n = 24 (10.04%)	n.a.
**Missing**	n = 12 (5.02%)	n.a.
**Gleason score**		
**2–6**	n = 150 (62.76%)	n.a.
**7**	n = 45 (18.82%)	n.a.
**8–10**	n = 29 (12.13%)	n.a.
**Missing**	n = 15 (6.27%)	n.a.
**PSA levels (ng/ml)**		
**≤4.0**	n = 1 (0.42%)	n.a.
**4.1–10**	n = 101 (42.26%)	n.a.
**10.1–20**	n = 65 (27.19%)	n.a.
**>20**	n = 42 (17.57%)	n.a.
**>1,000**	n = 2 (0.84%)	n.a.
**Missing**	n = 28 (11.72%)	n.a.

1 SD, standard deviation; n.a., not applicable.

### mtDNA Haplogroup Distribution in Patients with Sporadic Prostate Cancer

The frequencies of mitochondrial haplogroups did not differ significantly between the patients with prostate cancer and the control subjects (Table 2).

**Table 2:** Frequencies (%) of European mitochondrial haplogroups in cases in controls.

Haplogroup	Patients withprostate cancern = 239	Controlsn = 150	p-value^1^
**H**	45.6 (n = 109)	42.7 (n = 64)	0.570
**I**	0.8 (n = 2)	1.3 (n = 2)	0.637
**J**	7.9 (n = 19)	10.7 (n = 16)	0.362
**K**	6.3 (n = 15)	7.3 (n = 11)	0.684
**T**	5.9 (n = 14)	4.7 (n = 7)	0.613
**U**	16.3 (n = 39)	14.7 (n = 22)	0.663
**W**	1.7 (n = 4)	1.3 (n = 3)	0.791
**X**	1.3 (n = 3)	2 (n = 2)	0.562
**V**	0 (n = 0)	1.3 (n = 2)	0.073
**Others** 2	14.2 (n = 34)	14 (n = 21)	0.950

1 p-value: Pearson Chi-square.

2 Haplogroups that could not be assigned to one of the nine major European haplogroups (D, F, L, M, N, O, P and R).

**Figure 1:**
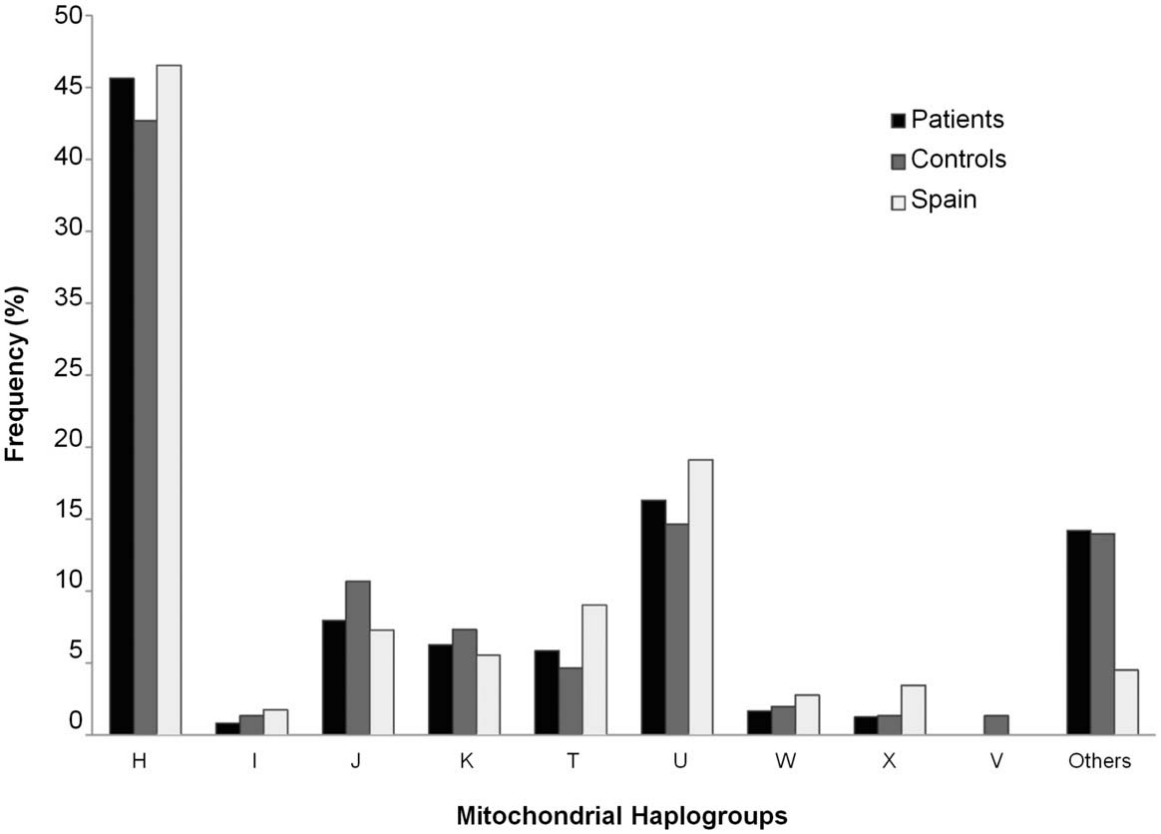
Bar graph representing the mitochondrial haplogroups in patients, controls, and the Spanish population.

These nine main haplogroups were also compared with the Spanish mitochondrial haplogroup distribution, as can be seen in Figure 1.

### CR Polymorphisms in Patients with Sporadic Prostate Cancer

The mitochondrial CR were sequenced and analyzed between nucleotide positions 16024 and 16365 (Tables S1 and S2). One hundred twenty-four homoplasmic polymorphisms and a heteroplasmic polymorphism (16169Y) were found among the 389 subjects compared to the revised Cambridge reference sequence [22]. Of the 125 polymorphisms detected, 13 were not listed in the Human Mitochondrial Genome Database [23], and 1 was neither listed in MITOMAP [24] nor the Human Mitochondrial Genome Database [23].

Eighteen of the 125 CR polymorphisms were detected at a frequency ≥4% in either the sporadic prostate cancer or the control group (Table 3). These were subjected to further statistical analysis. One of them, 16356C (p = 0.029) was found to have a significantly higher frequency in patients with sporadic prostate cancer compared to controls. Another polymorphism, 16278T has a p-value of 0.051 (Table 3). However, the significance was lost after correction for multiple comparisons by Bonferroni analysis, a significance level of <0.0004 is required.

**Table 3:** Frequencies (%) of most representative CR polymorphisms higher than 4% in either patients with prostate cancer or controls, and odds ratios (OR) for the association between genetic variation and disease state.

mtDNA CR polymorphisms	Frequency in patients (%)(Bootstrap)*	n^1^	Frequency in controls (%) (Bootstrap)*	n^1^	p-value^2^	OR^3^ (95%CI^4^)	p-value^5^	OR (95%CI)^5^	Frequency in Spain (%)(n = 630)	n^1^	Frequency in Europe (%)(n = 682)	n^1^	Allele count
T16311C	16.74 (12.1–21.5)	40	20.67(14.6–27.5)	31	0.329				16.19	102	15.84	108	40/199 C/T 31/119 C/T
T16126C	16.32(11.5–21.1)	39	18.67(13.0–25.3)	28	0.55				19.37	122	19.50	133	39/200 C/T 28/122 C/T
T16189C	14.64 (10.4–19.3)	35	12.67(7.7–18.7)	19	0.583				15.71	99	15.25	104	35/194 C/T 19/131 C/T
T16362C	12.55(8.5–17.1)	30	8(4.0–12.9)	12	0.159				9.37	59	3.23	22	30/209 C/T 12/138 C/T
C16270T	10.46(6.9–14.4)	25	12(7.0–18.0)	18	0.637				9.68	61	6.89	47	25/214 T/C 18/132 T/C
G16129A	9.62(6.1–13.4)	23	5.33(2.1–9.2)	8	0.128				6.83	43	10.70	73	23/216 A/G 8/142 A/G
C16069T	9.21(5.6–13.0)	22	10.67(6.0–16.1)	16	0.636				7.46	47	10.12	69	22/217 T/C 16/134 T/C
C16223T	9.21(5.6–13.0)	22	10(5.5–15.4)	15	0.795				12.38	78	15.54	106	22/217 T/C 15/135 T/C
T16298C	9.21(5.6–13.0)	22	7.3(3.5–12.2)	11	0.573				7.78	49	12.46	85	22/217 C/T 11/139 C/T
C16278T	8.37(5.1–12.1)	20	14(9.2–20.9)	21	0.051	1.882 (0.989–3.582)	0.090		10.48	66	4.25	29	20/219 T/C 21/129 T/C
T16304C	7.95(5.9–11.3)	19	9.33(5.1–14.5)	14	0.634				8.10	51	7.33	50	19/220 C/T 14/136 C/T
T16224C	7.53(4.6–11.1)	18	7.33(3.5–12.2)	11	0.942				6.03	38	9.68	66	18/221 C/T 11/139 C/T
C16294T	7.53(4.6–11.1)	18	6(2.6–10.1)	9	0.563				10.32	65	9.82	67	18/221 T/C 9/141 T/C
T16172C	5.86(3.2–9.1)	14	4(1.3–7.3)	6	0.419				4,44	28	6.01	41	14/225 C/T 6/144 C/T
C16192T	5.44(2.9–8.5)	13	7.33(3.5–12.2)	11	0.45				7.62	48	4.40	30	13/226 T/C 11/139 T/C
A16183C	5.19(2.6–8.0)	12	3.33(0.7–6.5)	5	0.428				6.98	44	2.05	14	12/227 C/A 5/145 C/A
G16145A	5.02(2.6–8.0)	12	2.67(0.6–5.7)	4	0.255				1.75	11	3.67	25	12/227 A/G 4/146 A/G
T16356C	4.6(2.1–7.5)	11	0.67(0.0–2.2)	1	0.029	0.139 (0.018–1.089)	0.041	0.639(0.435-3.878)	2.06	13	0.88	6	11/228 C/T 1/149 C/T

1 n: number of individuals with the respective polymorphism.

2 p-value: derived from Pearson chi-square.

3 OR: odds ratio.

4 CI: confidence interval.

5 p-value: adjusted for age.

* : Bootstrap for each percentage (patients and controls) with a CI of 95% calculated for 1,000 bootstrap samples.

The linkage disequilibrium analysis results show that some CR polymorphisms were out of balance (p = 0.05); the standardized disequilibrium values are shown in Table S3. The T16356C substitution associated with patients (p = 0.029) is linked to A16183C and T16189C (p<0.05). The mutation C16278T (p = 0.051) is associated with C16069T, T16126C, T16172C, T16189C, and C16223T (p<0.05).

### Analysis of Clinical Parameters

We also analyzed the relation between the clinical parameters associated with tumor invasiveness and progression (PSA levels, Gleason score, and T-stage) and the haplogroup frequencies, as far as CR polymorphism.

When the five most prevalent haplogroup frequencies were compared with the main clinical parameters used to diagnose prostate cancer (PSA levels, Gleason score, and T-stage), no significance differences were observed among prostate cancer patients (data not shown). Furthermore, when the clinical parameters were subdivided into categories: PSA levels (≤4.0, 4.1–10, 10.1–20, >20, >1,000), Gleason score (2–6, 7, 8–10), and T-stage (A, B, C, D), none of the haplogroups reached statistical significance (Table 4).

**Table 4:** Summary of statistical data on haplogroups in patients and clinical data.

		Haplogroup H		Haplogroup U		Haplogroup K		Haplogroup J		Haplogroup T	
		%	p-value	%	p-value	%	p-value	%	p-value	%	p-value
**Age**	≤55	7.1		8.8		8.3		9.1		9.1	
	56–60	10	0.431 (F)	5.9	0.431 (F)	8.3	0.431 (F)	18.2	0.431 (F)	36.4	0.431 (F)
	61–65	24.3	0.371 (MC)	11.8	0.371 (MC)	8.3	0.371 (MC)	18.2	0.371 (MC)	9.1	0.371 (MC)
	>65	58.6		73.5		75		54.5		45.5	
**Stage**	A	4.3		2.9		8.3		0		0	
	B	60.9	0.851 (F)	58.8	0.851 (F)	75	0.851 (F)	81.8	0.851 (F)	54.5	0.851 (F)
	C	24.6	0.837 (MC)	29.4	0.837 (MC)	8.3	0.837 (MC)	9.1	0.837 (MC)	18.2	0.837 (MC)
	D	10.1		8.8		8.3		9.1		27.3	
**PSA**	≤4.0	0.8		0		0		0		0	
	4.1–10	47.6		48.4		36.4		45.5		36.4	
	10.1–20	27	0.723 (F)	29	0.723 (F)	27.3	0.723 (F)	54.5	0.723 (F)	27.3	0.723 (F)
	>20	20.6	0.679 (MC)	19.4	0.679 (MC)	18.2	0.679 (MC)	0	0.679 (MC)	18.2	0.679 (MC)
	>1000	4		3.2		18.2		0		18.2	

Vr, versomility reason; cv, contingency value; F, Fisher; MC, Monte Carlo; %, % of haplogroup in patients.

When analyzing the CR polymorphisms and clinical parameters among patients, only significant differences were observed in Gleason score and T-stage. The variant G16129A was found in 8.79% of the patients with a Gleason score between 2–6, in 0.42% of the patients with a Gleason score of 7, and in 0.42% of the patients with a Gleason score of 8–10 (p = 0.007). Also, the mutation T16224C was found in 3.76% of the patients with a Gleason score between 2–6, in 2.09% of the patients with a Gleason score of 7, and in 0.41% of the patients with a Gleason score of 8–10 (p = 0.022). The T16311C substitution reached significance when compared to the four T-stage categories: 1.67% of patients in category A, 9.21% in B, 1.67% in C, and 1.67% in D (p = 0.046).

## Discussion

Human mitochondrial DNA is widely used as a tool in many fields, including evolutionary anthropology and population history, medical genetics, genetic genealogy, and forensic science [25]. The main role of mitochondria related to malignancy and cancer biology is likely due to their essential function in the generation of ATP and the regulation of apoptosis [8]. Because of the high mutation rate in the 1.1 kb non-coding control region (16024-576), which is about ten times higher than the mutation rate in the 15.5 kb coding region (bases 577–16023) [26], the non-coding region is relatively enriched in sequence variation, and haplogroup information can be obtained from this region. These haplogroups have been related to some oncological diseases, such as breast, colorectal, and thyroid [27] cancer, as well as other diseases, such as AIDS [28].

The most prevalent haplogroup in this population is H (45.6% in patients and 42.7% in controls), which is also the most widespread in Occidental Europe. We have also distinguished the other eight main haplogroups found in the European population (I, U, K, J, T, W, X, and V) [29] and, in a minority percentage, the haplogroups D, F, L, M, N, O, P, and R. As can be seen from the data, the haplogroups H, U, and W were more prevalent in patients than in controls, whereas the haplogroups I, J, and K were observed in controls (Table 2). Nevertheless, no significant differences in haplogroup frequencies between patients with sporadic prostate cancer and control subjects could be found, as also reported by Mueller et al. [21], who performed a case-control study of 304 patients with prostate carcinoma in Middle Europe. However, a study in a North American population of European descendent reported an elevated presence of the haplogroup U in prostate carcinoma patients [10]. One of the limitations of our study is the restricted sample size (239 patients and 150 controls).

Comparing the haplogroups in this study with that in the Spanish population [30], the percentages of mitochondrial haplogroups in Spain were found to be very similar to the data obtained in our analysis (46.53% H, 19.10% U, 9.03% T, 7.29% J, 5.56% K, and 1.74% I) (Figure 1).

With regard to the clinical characteristic analysis, no association was observed between mitochondrial haplogroup and age, PSA serum levels, Gleason score, or T-stage. However, when all clinical characteristics were categorized, no association was found with any haplogroups due to the fact that the limited number of patients enrolled was not enough for statistical analysis in small subgroups. However, the highest PSA levels (10.1–20, >20, and >1,000) and Gleason scores (7 and 8–10), and increased proportions of T-stage C were found in patients with the haplogroups H and U (Table 4).

The SNPs in the D-loop region have been examined in other tumor types (pancreatic, breast, or melanoma, among others) [31]–[33] as predictor factors of cancer risk. For this reason, we also compared the CR germinal polymorphisms of mtDNA in sporadic prostate cancer patients.

The T16356C variant has been previously related to glioblastoma [34] and breast cancer. In breast cancer, it has been included like a germ-line sequence variation [35]. Within our prostate cancer cohort, the T16356C mutation showed significantly elevated frequencies in prostate cancer patients compared to controls (p = 0.029). The C16278T polymorphism has also been previously related to cancer, particularly to breast cancer in Tan et al. [35], and to neurofibromatosis type-1 [36], as well as to other disorders, such as Parkinson’s disease [37]. Among our prostate cancer subjects, we have also found nearly significant elevated frequencies in prostate cancer patients compared to controls (p = 0.051). However, it is unclear whether these polymorphisms may be involved in tumor formation or progression, or the development of the other previously mentioned diseases.

The A16183C polymorphism has been established by MITOMAP [24] or the Human Mitochondrial Genome Database [23] to be related to prostate cancer disease [11], [38]. However, in our cohort, we did not find a significant association between this polymorphism and the disease (p = 0.428). In the analysis performed by Jeronimo et al., the position 16183 has been described as a mtDNA mutation in prostate cancer by the change A → G [38]; studies performed by Makiko et al. has associated this polymorphism to lung cancer patients [39].

We further analyzed the clinical parameters to determine if the CR polymorphisms in the patient cohort correlate with disease aggressiveness. The G16129A and T16224C polymorphisms were found to be associated with less aggressive Gleason scores (2–6) [40], with the scores closest to 2 as the least aggressive and those next to 10 as the most aggressive [41]. The G16129A substitution has been described in thyroid cancer and, in combination with T16362C, could have an effect on mtDNA replication and/or transcription, as well on increasing the risk of thyroid cancer [27]. On the other hand, the T16224C mutation has been described in patients with nonmelanoma skin cancer, but no association with the disease has been established [42]. On analyzing our results, no strong relation could be established because the Gleason score percentages of this variant were all below 5%. The T16311C variant was found at higher frequency in patients with T-stage B (also known as stage II), which includes cancers that have not spread outside the prostate (local cancer) [43], [44]. This polymorphism was previously described by Chen et al. [45] as a mtDNA mutation detected in the D-loop region in neoplastic lesions dissected from 16 prostatectomy specimens, but no information about clinical features were provided; it has also been found to be significantly associated with human colorectal cancer [46].

As only CR and mitochondrial haplogroups were analyzed, mitochondrial coding region polymorphisms cannot be excluded as possible mutations associated with prostate cancer [8], [47]. Somatic mutations in mitochondrial DNA (mtDNA) have been identified in various tumors, including breast cancer [48], squamous cell carcinoma [49], and prostate cancer [15], [50]. In squamous cell carcinoma, they were associated with better survival. However, in breast cancer somatic mutations are suggested to play a critical role in the progression of the cancer. Somatic mutations are frequent events in prostate cancer. Mutations mapping to mitochondrial tRNAs, ribosomal RNAs, and protein coding genes might impair processes that occur within the mitochondrial compartment (e.g., transcription, RNA processing, and translation) and might ultimately affect oxidative phosphorylation [15]. Germline and somatic mutations have also been described in patients with uterine fibroids [51] and prostate cancer [50], but there is not as much information on these as for somatic mutations in cancer. In this study, only blood genomic DNA samples were available, and no associations between somatic mutations could be determined.

In conclusion, our study confirms the lack of association between any mitochondrial haplogroup and sporadic prostate cancer.

## Methods

### Ethics Statement

This study complied with the Declaration of Helsinki. Informed consent was obtained from all subjects before they were entered into the study. The study and use of archive samples for this project were approved by the Ethics Committee of the University “Hospital Virgen de las Nieves,” Granada, Spain.

### Patients and Controls

Patients with sporadic prostate cancer were selected by a urologist, who also made notations about important parameters for prostate cancer, such as PSA level, T-score, Gleason score, and other information, including age, place of birth, and family history of prostate cancer. In brief, the eligible patients were adult males with a recent diagnosis of prostate cancer (n = 239). The clinical features of the patients selected for the study were histopathologically confirmed primary adenocarcinoma after abnormal serum PSA findings and/or lower urinary tract symptoms. Our patients were unrelated European men with a mean age of 67.4 years and a mean Gleason score of 7.0, which indicates a dominant growth pattern of the tumor [52]–[54]. Healthy unrelated European men (n = 150) from the same geographic area with no history of prostate cancer were enrolled as controls (no PSA levels were detected and clinical evolution over some years was followed to avoid including affected men with prostate cancer as controls). Controls belonged to the same age group as patients; they all were men with health problems like renal lithiasis or andrological problems, so PSA analysis was performed to dismiss a possible prostate cancer. They all have normal PSA values with blood levels below 4 ng/ml, as well as a normal rectal touch. Informed consent was obtained from all the subjects in this study, which was approved by the Ethics Committee of the hospital. Peripheral blood samples were drawn from all participants (n = 389) into tubes containing K3-EDTA.

### DNA Extraction and Genotyping

Genomic DNA from patients and controls was extracted from peripheral blood using the standard organic extraction procedure by phenol/chloroform/isoamyl alcohol and proteinase K, and purification with Microcon® 100 filters (Millipore). Mitochondrial DNA sequencing and haplotyping were performed using AmpliTaq Gold® PCR Master Mix (Applied Biosystems), which includes all of the chemical components, except the primers and template, necessary for PCR in a GeneAmp System 2400 thermal cycler. Each PCR reaction was performed in a total volume of 25 µL containing 1–2 ng/µL of DNA, 0.5 µL of each primer covering the HVI (nucleotide positions 16024–16365) region, and 12.5 µL of AmpliTaq Gold® PCR Master Mix (Applied Biosystems). The amplification conditions were 95 °C for 11 min followed by 32 cycles at 95 °C for 10 sec, at 60 °C for 30 sec, and at 72 °C for 30 sec. The amplicons were purified using Microcon® 100 filters (Millipore). Sequencing reactions were performed using ABI PRISM® BigDye Terminator v.1.1 Cycle Sequencing Ready Reaction Kits. Each sequencing reaction was performed in a total volume of 10 µL containing 3 µL of DNA, 3 µL of each specific primer, 2 µL of kit, and 2 µL of buffer from the ABI PRISM® BigDye Terminator v.3.1 kit. The thermal cycling conditions were 95 °C for 1 min followed by 25 cycles at 95 °C for 15 sec, at 50 °C for 1 sec, and at 60 °C for 2 min. The excess dye terminator was removed by gel filtration with Performa ® DTR Cartridges (EdgeBio) and analyzed using the automated DNA sequencer ABI PRISM® 3130 (Applied Biosystems). The results of the analysis were edited using the ABI PRISM® SeqScape® v.2.6 software.

### Haplotype Tagging and Statistical Analysis

Haplogroups are defined by CR polymorphisms and any giving range of the mitochondrial genome can be used for haplogroup classification, which is based on the phylogenetic stability of mtDNA polymorphisms. The assignment of haplogroups to the samples was done using online software for mitochondrial DNA, such as the Haplogroup Prediction Tool of “The Genographic Project” [55], Aplo haplogroup search [56], and mtDNA manager [57]. Furthermore, all the haplogroups in this study were confirmed by Web programs, such as HaploGrep [58] and Phylotree [25]. Obtained results were analyzed and revised by expertise personal in the area by the presence or lack of some positions in comparison with reference sequence and checked by the use of last version available at Phylotree [25].

Using the software package SPSS v.15.0 [59], we performed statistical analyses, including the χ^2^ test, Fisher’s exact test, Monte Carlo testing, and generated contingency tables. The frequencies of all mitochondrial haplogroups and CR polymorphisms in the prostate cancer patients and controls were tested for independency using Pearson chi-square statistics and Fisheŕs exact test as appropriate. These analyses were performed to test for independence between the haplogroup data and the clinical data, such as age (≤55, 56–60, 61–65, >65), Gleason score (2–6, 7, 8–10), T-stage (A, B, C, D), and PSA levels (≤4.0, 4.1–10, 10.1–20, >20, >1,000). All tests considered the nominal statistical significance (p-value) to be <0.05. Only CR variants with a frequency ≥4% in either the prostate cancer or the control group were subjected to further statistical analysis. Association of T16356C with the disease was adjusted for age by logistic regression analysis. For analysis of T16356C polymorphism significant p-value was corrected for multiple comparisons by Bonferroni analysis leading to a new required significance level of <0.0004 (number of comparisons = 125). Linkage disequilibrium analysis was performed between all pairs of CR polymorphisms using 10,000 steps in the Markov Chain and 1,000 dememorization steps. D, D′, and r2 coefficients were computed with a significance level of 0.05 using the Arlequin v3.5 software [60].

## Supporting Information

Table S1 Control region polymorphisms and haplogroup classification for 239 patients with sporadic prostate cancer.
(PDF)

Table S2 Control region polymorphisms and haplogroup classification for 150 controls with sporadic prostate cancer.
(PDF)

Table S3 Significant linkage disequilibrium for CR polymorphisms.
(PDF)
