# Supplementary material for: Mitochondrial Haplogroups and Polymorphisms Reveal No Association with Sporadic Prostate Cancer in a Southern European Population
Source: PLoS One. 2012 Jul 17;7(7):e41201. doi: 10.1371/journal.pone.0041201 (PMC3398884; doi:10.1371/journal.pone.0041201)
Supplement: Table S1 — Control region polymorphisms and haplogroup classification for 239 patients with sporadic prostate cancer. (PDF) [file pone.0041201.s001.pdf]

Table S1: Control region polymorphisms and haplogroup classification for 239 patients of sporadic prostate cancer.

Individuals	N	HAPLOGROUPS	HV1 Polymorphisms
**CP16/07, CP24/07, CP46/07, CP48/07, CP49/07, CP54/07, CP61/07, CP68/07, CP76/07, CP92/07, CP96/07, CP103/07, CP110/07, CP121/07, CP122/07, CP125/07, CP14/08, CP16/08, CP22/08, CP29/08, CP37/08, CP52/08, CP59/08, CP64/08, CP65/08, CP71/08**	26	H	No polymorphisms
**CP78/07, CP117/07, CP118/07, CP01/08, CP20/08, CP36/08, CP48/08, CP61/08, CP66/08, CP68/08, CP33/08**	9	H+16362	16362C
**CP23/07, CP58/07, CP87/07, CP124/07, CP35/08, CP40/08, CP78/08, CP102/08**	8	HV0	16298C
**CP35/07, CP104/07, CP115/07, CP18/08, CP62/08, CP89/08**	6	H1j+16129	16129A
**CP119/07**	5	H5	16304C
**CP18/07, CP55/07, CP83/07, CP127/07, CP06/08**	5	J	16069T 16126C
**CP42/07, CP43/07, CP91/07, CP95/07, CP05/08**	5	K	16224C 16311C
**CP06/07, CP07/07, CP10/08, CP49/08**	4	J2a1a	16069T 16126C 16145A 16231C 16261T
**CP04/07, CP39/08, CP76/08, CP79/08**	4	U4	16356C
**CP51/07, CP25/08, CP91/08**	3	H2a2b	16291T
**CP22/07, CP70/07, CP77/07**	3	H2b	16311C
**CP80/07, CP85/07, CP105/07**	3	H5	16304C
**CP128/07, CP38/08, CP41/08**	3	K1a5	16224C 16311C 16362C
**CP83/08, CP85/08, CP105/08**	3	L2-6	16187T 16298C 16311C
**CP45/07, CP58/08, CP99/08**	3	T	16294T
**CP30/07, CP31/07**	2	H1b	16189C 16356C 16362C
**CP08/08, CP30/08**	2	H20a	16218T 16328A 16362C
**CP34/08, CP43/08**	2	H2b	16311C
**CP116/07, CP123/07**	2	H5	16294T 16304C 16320T
**CP36/07**	2	H6a1a1a	16311C 16362C
**CP93/07, CP98/07**	2	HV0	16129A 16298C
**CP42/08, CP109/08**	2	HV4b	16069T 16126C
**CP82/07, CP84/07**	2	J2a1	16069T 16126C 16231C
**CP84/08, CP96/08**	2	L3e2	16223T 16292T 16320T
**CP09/08, CP15/08**	2	R0a	16126C 16362C
**CP17/07, CP101/07**	2	R8a1+16093	16093C
**CP50/07, CP112/07**	2	R8b2	16172C
**CP89/07, CP108/07**	2	T1a	16126C 16163G 16186T 16189C 16294T
**CP12/07, CP26/08**	2	U2d	16051G 16183C 16189C 16234T 16266T 16294T
**CP27/07, CP120/07**	2	U3	16343G
**CP26/07, CP100/07**	2	U5a1+16192!	16256T 16270T
**CP40/07, CP04/08**	2	U5a1d2a	16145A 16172C 16189C 16243C 16256T 16270T

Individuals	N	HAPLOGROUPS	HV1 Polymorphisms
**CP59/07, CP79/07**	2	U6a	16172C 16219G 16278T
**CP112/08**	1	D4m2	16042A
**CP36/08**	1	H+16362	16189C 16298C 16362C
**CP33/08**	1	H1+16189	16189C
**CP10/07**	1	H11a1	16278T 16311C
**CP11/07**	1	H11a1	16278T
**CP03/07**	1	H1a	16162G
**CP64/07**	1	H1b	16189C 16255A 16356C 16362C
**CP23/08**	1	H1b	16182C 16183C 16189C 16356C
**CP45/08**	1	H1j+16129	16129A 16298C 16362C
**CP110/08**	1	H1j+16129	16129A 16223T
**CP46/08**	1	H24	16129A 16134T 16240G 16286A 16293G 16356C
**CP82/08**	1	H24	16293G 16311C
**CP90/07**	1	H2a1	16192T 16354T
**CP94/07**	1	H2a2	16150T 16320T
**CP126/07**	1	H2a2	16254R
**CP32/08**	1	H2a2	16247T 16342C
**CP107/08**	1	H2a2	16076T 16261T
**CP102/07**	1	H2a2b1	16235G 16291T
**CP113/07**	1	H2b	16248T 16311C
**CP97/08**	1	H2b	16311C 16355T
**CP98/08**	1	H2b	16266T 16304C 16311C
**CP07/08**	1	H5	16290T 16304C
**CP19/07**	1	H6b	16180G 16300G 16325C 16362C
**CP39/07**	1	H7a1	16129A 16261T
**CP21/07**	1	HV0	16234T 16298C
**CP32/07**	1	HV0	16234T 16298C
**CP33/07**	1	HV0	16129A 16271A 16298C
**CP75/07**	1	HV0	16234T 16298C
**CP98/07**	1	HV0	16129A 16298C
**CP57/08**	1	HV0	16129A 16271A 16298C
**CP92/08**	1	HV2a	16335G
**CP53/08**	1	HV4b	16069T 16126C 16291T 16293G
**CP69/08**	1	HV4b	16069T 16126C 16278T 16362C
**CP02/07**	1	I	16129A 16223T
**CP80/08**	1	I	16129A 16148T 16223T
**CP34/07**	1	J	16069T 16114T 16126C 16259A
**CP65/07**	1	J	16069T 16126C 16182C 16183C 16189C 16290T 16362C
**CP99/07**	1	J	16069T 16093C 16126C 16278T
**CP60/08**	1	J1d	16069T 16126C 16193T
**CP108/08**	1	J1d	16069T 16126C 16193T 16362C
**CP63/07**	1	J2b1a	16069T 16126C 16193T 16278T
**CP66/07**	1	J2b1a	16069T 16126C 16145A 16193T 16278T 16344T
**CP24/08**	1	JT	16126C
**CP47/08**	1	K	16224C 16270T 16311C
**CP74/08**	1	K	16092C 16184A 16224C 16311C

Individuals	N	HAPLOGROUPS	HV1 Polymorphisms
**CP20/07**	1	K1a1	16093C 16224C 16290T 16311C
**CP44/08**	1	K2a1a	16224C 16311C
**CP63/08**	1	K2a1a	16224C 16298C
**CP29/07**	1	K2b1	16224C 16270T 16362C
**CP109/07**	1	K2c	16192T 16224C 16311C
**CP72/07**	1	L0a1b	16129C 16148T 16168T 16172C 16187T 16188G 16189C 16214T 16223T 16230G 16278T 16293G 16311C 16320T
**CP38/07**	1	L1b	16126C 16187T 16189C 16223T 16264T 16270T 16278T 16293G 16311C
**CP67/07**	1	L1b	16126C 16187T 16189C 16223T 16264T 16270T 16278T 16293G 16311C 16317G
**CP81/08**	1	L2a1c5	16129A 16223T 16278T 16294T 16309G
**CP70/08**	1	L3e5	16041G 16172C 16223T
**CP74/07**	1	L3h2	16184T 16304C
**CP129/07**	1	L3i2	16260T 16311C
**CP56/08**	1	L3x1+16311	16169T 16290T 16311C
**CP75/08**	1	M1	16129A 16183C 16189C 16223T 16249C 16311C
**CP44/07**	1	M25	16129A 16223T 16304C
**CP100/08**	1	M2a1a3+16093	16093C 16224C 16270T 16311C 16319A
**CP28/07**	1	M5a1	16129A 16223T 16291T 16298C
**CP111/07**	1	M5c2	16240G
**CP86/08**	1	N	16223T
**CP56/07**	1	N1b1+16129	16086C 16129A 16145A 16176G 16223T
**CP47/07**	1	P3a	16145A
**CP52/07**	1	R11	16189C 16311C
**CP77/08**	1	R23	16256T 16270T
**CP11/08**	1	R5a	16266T 16304C 16311C 16362G 16363T
**CP73/08**	1	R8a1+16093	16093C 16129A
**CP104/08**	1	R8a1+16093	16093C 16224C 16290T 16311C
**CP106/08**	1	R8a1+16093	16093C 16126C
**CP93/08**	1	T	16126C 16292T 16294T
**CP05/07**	1	T1a	16126C 16163G 16186T 16189C 16294T 16335G
**CP12/08**	1	T1a	16126C 16163G 16186T 16189C 16234T 16294T
**CP71/07**	1	T2a1b	16126C 16287T 16294T 16296T 16324C
**CP09/07**	1	T2b	16126C 16207G 16294T 16296T 16304C
**CP31/08**	1	T2b	16126C 16145A 16294T 16296T 16304C
**CP69/07**	1	T2b3a	16126C 16183C 16189C 16292T 16294T 16296T 16304C
**CP67/08**	1	T2c	16126C 16291T 16292T 16296T
**CP19/08**	1	T2f	16126C 16189C 16196A 16294T 16296T
**CP60/07**	1	U2e1	16051G 16111T 16129C 16145A 16189C 16362C
**CP87/08**	1	U2e1	16051G 16129C 16183C 16189C 16362C
**CP55/08**	1	U4	16183C 16189C 16356C
**CP90/08**	1	U4a1	16134T 16240G 16286A 16293G 16356C
**CP25/07**	1	U5	16192T 16270T 16319A
**CP28/08**	1	U5	16074G 16189C 16192T 16270T

Individuals	N	HAPLOGROUPS	HV1 Polymorphisms
**CP13/07**	1	U5a	16192T 16256T 16270T
**CP21/08**	1	U5a1c	16227G 16256T 16270T 16320T
**CP01/07**	1	U5b1b1+16192!	16183C 16189C 16270T
**CP08/07**	1	U5b1b1+16192!	16111T 16189C 16270T
**CP62/07**	1	U5b1b1+16192!	16189C 16270T
**CP88/08**	1	U5b1b1+16192!	16093C 16183C 16189C 16270T
**CP73/07**	1	U5b1b1b	16183C 16189C 16270T 16320T 16357C
**CP94/08**	1	U5b1b1b	16192T 16256T 16270T 16286T 16320T
**CP17/08**	1	U5b1c	16051G 16172C 16189C 16192T 16270T 16311C
**CP81/07**	1	U5b1d	16270T
**CP130/07**	1	U5b1d	16270T 16274A 16311C 16325C
**CP27/08**	1	U5b1d	16129A 16192T 16270T 16325C
**CP50/08**	1	U5b1d	16227G 16256T 16270T 16316G 16320T
**CP41/07**	1	U5b2a1b	16325C
**CP14/07**	1	U6a	16145A 16172C 16219G 16235A 16278T
**CP15/07**	1	U6a	16172C 16219G 16278T
**CP37/07**	1	U6a	16172C 16192T 16219G 16278T
**CP88/07**	1	U6a	16172C 16219G 16224C 16234T 16278T
**CP13/08**	1	U6a	16092C 16172C 16219G 16278T
**CP106/07**	1	W1e	16223T 16292T 16295T
**CP53/07**	1	W6	16192T 16223T 16292T 16325C 16342C
**CP57/07**	1	W6	16192T 16223T 16292T 16325C
**CP51/08**	1	W6	16093C 16192T 16223T 16292T 16325C
**CP86/07**	1	X	16183C 16189C 16223T 16278T
**CP101/08**	1	X	16189C 16192T 16223T 16278T 16304C
**CP54/08**	1	X2j	16179T 16188T 16189C 16223T 16278T
