# Supplementary material for: Mitochondrial Haplogroups and Polymorphisms Reveal No Association with Sporadic Prostate Cancer in a Southern European Population
Source: PLoS One. 2012 Jul 17;7(7):e41201. doi: 10.1371/journal.pone.0041201 (PMC3398884; doi:10.1371/journal.pone.0041201)
Supplement: Table S2 — Control region polymorphisms and haplogroup classification for 150 controls with sporadic prostate cancer. (PDF) [file pone.0041201.s002.pdf]

Table S2: Control region polymorphisms and haplogroup classification for 150 controls.

Individuals	N	HAPLOGROUPS	HV1 Polymorphisms
**CP119/08, CP129/08, CP144/08, CP148/08, CP150/08, CP155/08, CP159/08, CP166/08, CP170/08, CP185/08, CP196/08, CP214/08, CP227/08, CP234/08, CP240/08, CP246/08, CP259/08,**	18	H	
**CP136/08, CP171/08, CP176/08, CP189/08, CP244/08, CP268/08**	6	H5	16304C
**CP116/08, CP130/08, CP164/08, CP218/08**	4	H+16362	16362C
**CP163/08, CP168/08, CP188/08**	4	K	16224C 16311C
**CP128/08, CP199/08, CP241/08**	3	H1j+16129	16129A
**CP117/08, CP125/08, CP162/08**	3	H2b	16311C
**CP175/08, CP198/08, CP222/08**	3	HV0	16298C
**CP126/08, CP151/08, CP258/08**	3	J	16069T 16126C
**CP133/08, CP140/08, CP169/08**	3	J2b1a	16069T 16126C 16278T
**CP139/08, CP186/08, CP195/08, CP223/08**	3	K1a1	16093C 16224C 16311C
**CP132/08, CP191/08,**	3	U5b1d	16270T
**CP226/08, CP239/08**	2	H+16192	16192T
**CP137/08, CP167/08**	2	H1+16189	16183C 16189C
**CP212/08, CP250/08**	2	H11a1	16278T
**CP216/08, CP257/08**	2	J2b1a	16069T 16126C 16193T 16278T
**CP123/08, CP206/08**	2	L1b	16126C 16187T 16189C 16223T 16264T 16270T 16278T 16311C 16360T
**CP143/08, CP205/08**	2	T2b3a	16126C 16183C 16189C 16292T 16294T 16296T 16304C
**CP142/08, CP236/08**	2	U5	16192T 16270T 16319A
**CP255/08**	1	D1f	16142T 16223T 16325C 16362C
**CP203/08**	1	F1a2	16172C 16304C 16311C
**CP152/08**	1	H+16362	16102G 16104A 16362C
**CP248/08**	1	H+16362	16170G 16362C
**CP243/08**	1	H1a1	16162G 16209C 16239T
**CP127/08**	1	H1f	16093C 16189C
**CP146/08**	1	H1k	16189C 16219G 16278T 16290T 16311C
**CP181/08**	1	H1x	16037G
**CP135/08**	1	H20	16218T 16298C
**CP235/08**	1	H20	16218T
**CP141/08**	1	H20a	16218T 16328A 16362C
**CP245/08**	1	H24	16213A 16293G
**CP215/08**	1	H27	16129A 16316G
**CP251/08**	1	H2a1	16354T
**CP242/08**	1	H2a2	16239T 16242T
**CP118/08**	1	H2a2b	16291T
**CP217/08**	1	H5	16294T 16304C 16320T

Individuals	N	HAPLOGROUPS	HV1 Polymorphisms
**CP149/08**	1	H7a1	16261T
**CP197/08**	1	HV0	16240G 16298C
**CP230/08**	1	HV0	16075C 16189C 16298C
**CP247/08**	1	HV0	16180G 16189C 16298C
**CP202/08**	1	HV13	16294T 16357C 16358T 16363T
**CP253/08**	1	HV2a	16335G
**CP265/08**	1	I	16086C 16129A 16223T
**CP120/08**	1	I5a	16129A 16148T 16223T 16291T 16298C
**CP190/08**	1	J	16069T 16126C 16278T 16362C
**CP228/08**	1	J1b1a1	16069T 16126C 16145A 16172C 16222T
**CP114/08**	1	J1c+16261	16069T 16126C 16261T
**CP174/08**	1	J2a1a	16069T 16126C 16140C 16145A 16231C 16261T
**CP208/08**	1	J2a1a	16069T 16126C 16145A 16231C 16261T 16311C
**CP219/08**	1	J2b	16069T 16126C 16179A 16193T 16278T 16311C
**CP156/08**	1	J2b1a	16069T 16126C 16193T 16250S 16278T
**CP264/08**	1	J2b1a	16069T 16126C 16193T 16278T 16311C
**CP113/08**	1	K	16124C 16224C 16271C 16311C
**CP179/08**	1	K	16224C 16235G 16311C
**CP145/08**	1	K1a1	16093C 16192T 16224C 16239T 16311C
**CP201/08**	1	K1b1a2	16093C 16224C 16311C 16318T 16319A
**CP157/08**	1	L1b	16126C 16187T 16189C 16223T 16264T 16270T 16278T 16293G 16311C
**CP221/08**	1	L1b1	16126C 16187T 16189C 16223T 16264T 16270T 16278T 16311C
**CP154/08**	1	L3a	16316G
**CP124/08**	1	L3h1	16223T 16311C
**CP231/08**	1	L3h1b1a	16179T 16223T 16243C 16256A 16284G 16311C 16320T
**CP262/08**	1	L3i2	16260T 16311C
**CP252/08**	1	L3x	16169T
**CP261/08**	1	M18	16223T 16318T
**CP256/08**	1	M5a1	16129A 16223T 16291T 16298C
**CP187/08**	1	M5c2	16240G
**CP260/08**	1	M65a	16289G
**CP122/08**	1	N1b	16145A 16176G 16223T
**CP225/08**	1	O	16213A
**CP204/08**	1	P+16176	16176T
**CP220/08**	1	P+16176	16176T 16219G
**CP200/08**	1	R0a	16126C 16362C
**CP232/08**	1	R0a	16126C 16209C 16362C
**CP173/08**	1	T1a	16126C 16163G 16186T 16189C 16294T
**CP165/08**	1	T2b	16126C 16241G 16294T 16296T 16304C
**CP177/08**	1	T2b	16126C 16294T 16296T 16304C
**CP131/08**	1	T2b+16296!	16126C 16147T 16294T 16297C 16304C
**CP134/08**	1	T2c	16126C 16292T 16294T 16296T

Individuals	N	HAPLOGROUPS	HV1 Polymorphisms
**CP172/08**	1	U2	16051G
**CP115/08**	1	U3	16343G
**CP183/08**	1	U3	16343G 16357C 16358T 16363T
**CP238/08**	1	U3	16260T 16343G
**CP194/08**	1	U4	16356C
**CP266/08**	1	U5	16192T 16270T
**CP229/08**	1	U5a	16192T 16256T 16270T 16311C
**CP209/08**	1	U5a1+16192!	16256T 16270T
**CP233/08**	1	U5a1+16192!	16227G 16256T 16270T 16316G
**CP237/08**	1	U5a1b1	16192T 16256T 16270T 16291T
**CP254/08**	1	U5a1c	16180G 16192T 16256T 16270T 16320T
**CP224/08**	1	U5b1+16189	16189C 16192T 16270T
**CP153/08**	1	U5b1b1+16192!	16172C 16183C 16189C 16270T 16274A 16311C 16325C
**CP207/08**	1	U5b3	16192T 16270T 16304C
**CP192/08**	1	U6a	16172C 16219G 16278T
**CP249/08**	1	U6a	16169Y 16172C 16219G 16278T
**CP147/08**	1	U6a'b'd+16311	16172C 16174T 16219G 16311C
**CP210/08**	1	V7a	16153A 16186T 16298C
**CP267/08**	1	V7a	16153A 16189C 16298C
**CP138/08**	1	W	16223T 16234T 16292T
**CP211/08**	1	W	16223T 16292T
**CP121/08**	1	X	16189C 16223T 16278T
**CP178/08**	1	X2h	16129A 16189C 16278T 16311C 16362C
**CP193/08**	1	X2h	16189C 16278T 16311C
