# Supplementary material for: Mitochondrial Haplogroups and Polymorphisms Reveal No Association with Sporadic Prostate Cancer in a Southern European Population
Source: PLoS One. 2012 Jul 17;7(7):e41201. doi: 10.1371/journal.pone.0041201 (PMC3398884; doi:10.1371/journal.pone.0041201)
Supplement: Table S3 — Significant linkage disequilibrium for CR polymorphisms. (PDF) [file pone.0041201.s003.pdf]

Table S3: Table of significant linkage disequilibrium of CR polymorphisms.

Locus	16069	16126	16129	16145	16172	16183	16189	16192	16223	16224	16270	16278	16294	16298	16304	16311	16356	16362
16069		p=0.000<br>r2=0.499	-	p=0.000<br>r2=0.070	-	-	-	-	-	-	-	p=0.0002<br>r2=0.0449	-	-	p=0.0199<br>r2=0.0105	p=0.0072<br>r2=0.0167	-	-
16126	p=0.000<br>r2=0.499		p=0.0018<br>r2=0.022	p=0.0009<br>r2=0.039	p=0.0197<br>r2=0.011	-	-	p=0.0198<br>r2=0.0111	-	p=0.0046<br>r2=0.0188	-	p=0.000<br>r2=0.0480	p=0.000<br>r2=0.0988	-	-	-	-	-
16129	-	p=0.0018<br>r2=0.022		-	-	-	-	-	p=0.000<br>r2=0.0659	-	-	-	-	p=0.0006<br>r2=0.0346	-	-	-	-
16145	p=0.000<br>r2=0.070	p=0.0009<br>r2=0.039	-		p=0.0039<br>r2=0.0222	-	-	-	-	-	-	-	-	-	-	-	-	-
16172	-	p=0.0197<br>r2=0.011	-	p=0.0039<br>r2=0.0222		-	-	-	-	-	-	p=0.000<br>r2=0.1351	-	-	-	-	-	-
16183	-	-	-	-	-		p=0.000<br>r2=0.2886	-	-	-	-	-	p=0.0038<br>r2=0.0412	-	-	-	p=0.0110<br>r2=0.0132	-
16189	-	-	-	-	-	p=0.000<br>r2=0.2886		-	p=0.0024<br>r2=0.0319	p=0.0229<br>r2=0.0136	p=0.000<br>r2=0.0785	p=0.0004<br>r2=0.0415	p=0.0002<br>r2=0.0400	-	-	-	p=0.0136<br>r2=0.0243	-
16192	-	p=0.0198<br>r2=0.0111	-	-	-	-	-		-	-	p=0.000<br>r2=0.1006	-	-	-	-	-	-	-
16223	-	-	p=0.000<br>r2=0.0659	-	-	-	p=0.0024<br>r2=0.0319	-		-	-	p=0.000<br>r2=0.0566	-	-	-	-	-	-
16224	-	p=0.0046<br>r2=0.0188	-	-	-	-	p=0.0229<br>r2=0.0136	-	-		-	-	-	-	-	p=0.000<br>r2=0.2992	-	-
16270	-	-	-	-	-	-	p=0.000<br>r2=0.0785	p=0.000<br>r2=0.1006	-	-		-	-	-	-	-	-	-
16278	p=0.0002<br>r2=0.0449	p=0.000<br>r2=0.0480	-	-	p=0.000<br>r2=0.1351	-	p=0.0004<br>r2=0.0415	-	p=0.000<br>r2=0.0566	-	-		-	-	-	-	-	-
16294	-	p=0.000<br>r2=0.0988	-	-	-	p=0.0038<br>r2=0.0412	p=0.0002<br>r2=0.0400	-	-	-	-	-		-	p=0.000<br>r2=0.1052	p=0.0027<br>r2=0.0185	-	-
16298	-	-	p=0.0006<br>r2=0.0346	-	-	-	-	-	-	-	-	-	-		-	-	-	-
16304	p=0.0199<br>r2=0.0105	-	-	-	-	-	-	-	-	-	-	-	p=0.000<br>r2=0.1052	-		-	-	p=0.0020<br>r2=0.0062
16311	p=0.0072<br>r2=0.0167	-	-	-	-	-	-	-	-	p=0.000<br>r2=0.2992	-	-	p=0.0027<br>r2=0.0185	-	-		-	-
16356	-	-	-	-	-	p=0.0110<br>r2=0.0132	p=0.0136<br>r2=0.0243	-	-	-	-	-	-	-	-	-		-
16362	-	-	-	-	-	-	-	-	-	-	-	-	-	-	p=0.0020<br>r2=0.0062	-	-	

Table of significant linkage disequilibrium: Test of linkage disequilibrium for all pairs of loci (Slatkin, M. 1994a.,Slatkin, M. and Excoffier, L. 1996).

In green,all those positions that are out of balance(significance level=0.05). p= p-value; r2= standardized disequilibrium values.Nº of steps=10000,demorization steps=1000
